# Climate change awareness and risk perceptions in the coastal marine ecosystem of Palawan, Philippines

**DOI:** 10.14324/111.444/ucloe.000054

**Published:** 2023-01-26

**Authors:** Lutgardo B. Alcantara, Lota A. Creencia, John Roderick V. Madarcos, Karen G. Madarcos, Jean Beth S. Jontila, Fiona Culhane

**Affiliations:** 1 College of Fisheries and Aquatic Sciences, Western Philippines University, Puerto Princesa City, Philippines; 2 School of Biological and Marine Science, University of Plymouth, Plymouth, UK

**Keywords:** climate change awareness, risk perception, exposure, experience, impact, policy

## Abstract

Understanding coastal communities’ awareness and risk perceptions of climate change impact is essential in developing effective risk communication tools and mitigation strategies to reduce the vulnerability of these communities. In this study, we examined coastal communities’ climate change awareness and risk perceptions of climate change impact on the coastal marine ecosystem, sea level rise impact on the mangrove ecosystem and as a factor affecting coral reefs and seagrass beds. The data were gathered by conducting face-to-face surveys with 291 respondents from the coastal areas of Taytay, Aborlan and Puerto Princesa in Palawan, Philippines. Results showed that most participants (82%) perceived that climate change is happening and a large majority (75%) perceived it as a risk to the coastal marine ecosystem. Local temperature rise and excessive rainfall were found to be significant predictors of climate change awareness. Sea level rise was perceived by most participants (60%) to cause coastal erosion and to affect the mangrove ecosystem. On coral reefs and seagrass ecosystems, anthropogenic drivers and climate change were perceived to have a high impact, while marine livelihoods had a low impact. In addition, we found that climate change risk perceptions were influenced by direct experiences of extreme weather events (i.e., temperature rise and excessive rainfall) and climate-related livelihood damages (i.e., declining income). Climate change risk perceptions were also found to vary with household income, education, age group and geographical location. The results suggest that addressing poverty and effectively communicating climate change risks can improve climate change awareness and risk perceptions.

## Introduction

Climate change is the challenge of our generation. Its impacts are already seen in human health, agriculture, water resources, food safety, food security and coastal and marine ecosystems [1–5]. In coastal and marine ecosystems, climate change is causing two important impacts: sea level rise and changing ocean chemistry [6,7]. Thermal expansion brought on by ocean warming and land-based ice melting, such as glaciers and ice sheets, is the main factor contributing to sea level rise. Rising sea levels are expected to have the greatest influence on the distribution and condition of the mangrove ecosystem in the future [8,9]. Meanwhile, changes in ocean chemistry are caused by anthropogenic climate drivers, including increasing amounts of greenhouse gases and aerosols [10]. Because of increased greenhouse gas concentrations, the ocean’s sea surface temperature is rising and making the oceans more acidic, increasing the risks of coral bleaching, leading to coral death and losing critical habitats for other species [11,12]. These impacts of climate change which result in the loss of marine diversity and the degradation of coastal marine ecosystems are relatively well known [13,14]. However, coastal communities may perceive these impacts differently, which necessitates further investigation.

In the Philippines, the serious impacts of climate change are becoming more apparent – thus, the need for proactive mitigation and adaptation approaches has become an urgent public concern. The Philippines is one of the most vulnerable countries to sea level rise and its impacts due to its numerous low-lying coastal areas. Seven out of the 25 cities globally most vulnerable to a 1-m sea level rise are in the Philippines [15]. Based on the Marine Geological Survey Division report, from 1992 to 2011, the rate of sea level rise in the Philippines was 5.8 (± 0.6) mm per year [16]. This is faster compared to the global rate of sea level rise averages of 3.3 (± 0.4) mm per year [17]. At the current rate of sea level rise, by 2100 it would lead to the inundation of more than 167,000 ha of coastal land (about 0.6% of the country’s total area) and 171 towns, as well as the displacement of 13.6 million Filipinos [18]. In the 2015 simulation, Palawan is one of the Philippine provinces most vulnerable to coastal flooding due to its low coastline elevation zones [19,20]. With a 1-m sea level rise, 6428.16 ha of land is expected to be inundated in the province [19]. Thankfully, Palawan’s selection as a UNESCO Biosphere Reserve (BR) can help lessen the effects of climate change and spur efforts to mitigate and adapt to climate change [21].

Previous research in Palawan has explored adaptation strategies for enhancing climate resilience at the local level [22], assessed long-term climate variability’s effects on coral reefs’ biophysical conditions [23] and studied fishers’ perceptions and adaptation capacities [24]. Further research into community awareness and risk perceptions can give us a clearer picture on which to base conservation decision-making and environmental management, which will help the province better mitigate and adapt to the effects of climate change [25]. Additionally, this could lead to greater participation, more effective management practices that meet the capabilities of the concerned stakeholders, and eventually, faster restoration of maritime resources [26].

Studies have shown that people’s climate change awareness and risk perceptions vary widely and are influenced by various factors [27]. In Asia, the most important indicator of risk perception of climate change impacts is local temperature change [27,28], whereas globally, climate change awareness is determined by educational attainment [27]. Furthermore, personal experiences of other extreme weather events and impacts of climate change also influence climate change risk perceptions [29–31], as well as socio-demographic characteristics, which include gender, income [27,28], age [32], geographical location [33,34] and occupation [28]. Although several studies have been conducted globally to evaluate how these factors influence climate change awareness and risk perceptions, none have been done so far in the coastal communities of Palawan, Philippines, particularly on the perceived impact on the mangrove, seagrass and coral reef ecosystems.

The current study focuses on climate change awareness and risk perceptions of the impacts on coastal communities caused by sea level rise in the mangrove ecosystem, as well as the perceived impacts of climate change and anthropogenic drivers on coral reefs and seagrass beds [27,35]. This is a part of a more extensive survey conducted as part of the Global Challenges Research Fund (GCRF) Blue Communities project, which intends to investigate the well-being benefits and risks of coastal living in and around UNESCO BRs and Marine Protected Areas (MPAs) across Southeast Asia. The study approach was patterned with the ecosystems-enriched Drivers, Pressures, State, Exposure, Effects, Actions (eDPSEEA) model, which recognises the convergence between the idea of ecosystem services, which gives the value of ecosystems a human health and well-being slant while also emphasising the health of the environment, and the growing calls for ‘ecological public health’ as a response to global environmental concerns that are currently permeating the discourse in public health [36]. Specifically, the following are the objectives that this research attempted to address: (i) whether the participants are aware that climate is changing or not; (ii) whether they have experience of climate change impacts or not; (iii) whether climate change and sea level rise affect the coastal and mangroves ecosystems; (iv) whether climate change, anthropogenic drivers and marine livelihood affect the state of coral reefs and seagrass beds. The results of this study will contribute to the knowledge gap in understanding the climate change awareness and risk perceptions of the coastal communities to design more effective mitigation measures to address climate change impacts at the local level and for policies, programmes and activities aimed at building resilience to climate change and managing marine resources.

## Materials and methods

### Study area and sample

The Palawan province, known as the ‘last ecological frontier’ of the Philippines, is an archipelago composed of the main island and more than 1700 islands [21]. Its coastal marine ecosystems include coral reefs, seagrass meadows, mangroves and several marine mammals [21]. The province was declared as Mangrove Reserve Swamp in 1981 under Presidential Proclamation No. 2152 for having the most extensive remaining mangrove forest in the country, which was estimated at 63,532 ha in 2010 [37]. In 1991, it was also declared by UNESCO as a BR to serve as a learning area to promote sustainable development and conservation of biodiversity [21]. The projected population of Palawan in 2022 is 1,254,111 [38]. The primary economic activities are agriculture, fisheries, tourism, on-shore and off-shore mining, gathering of minor forest products and pearl farming [21].

**Figure 1:**
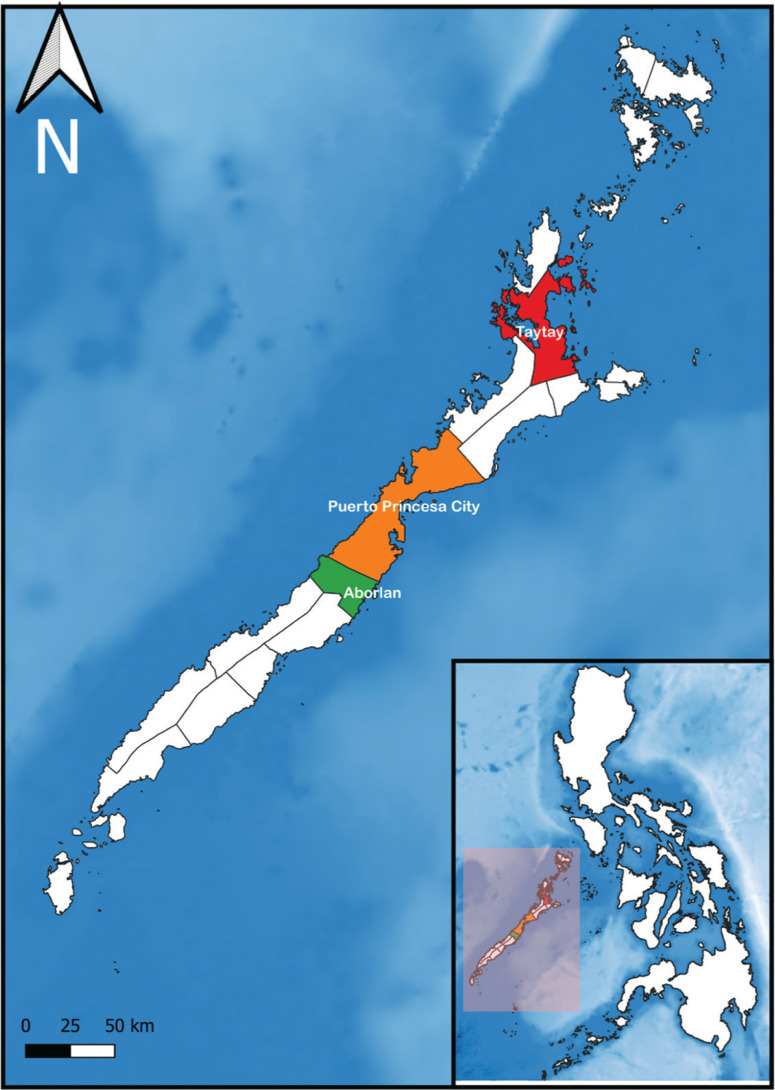
Map of Palawan showing an inset of the Philippines, with Palawan highlighted with a light red shade. Aborlan, Puerto Princesa City and Taytay are highlighted in green, orange and red colours, respectively. (Source: Authors, 2022.)

**Figure A1:**
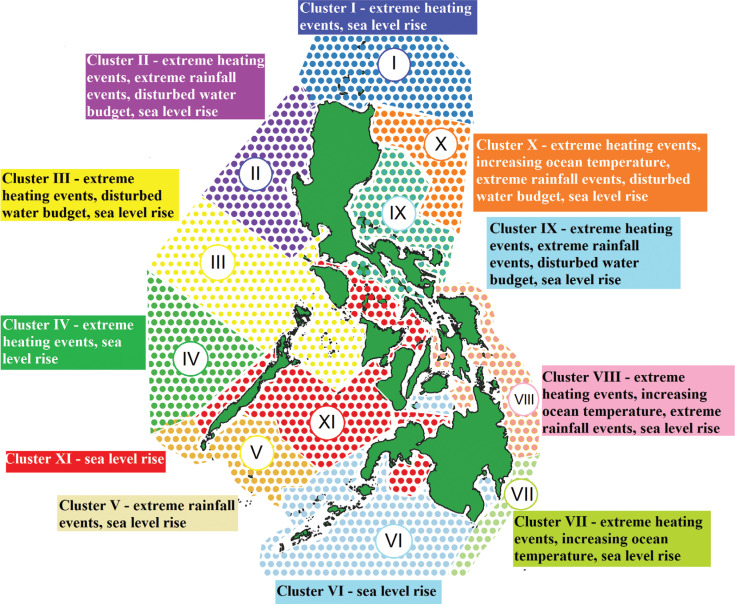
Philippine Exposure map on Climate Change (Source: Department of Environment and Natural Resources, Philippines, 2012).

The three study areas are Aborlan, a coastal municipality located in the southern part of the province; Puerto Princesa City, a highly urbanised coastal city, located in the central part; and Taytay, a coastal municipality located in the northern part (Fig. 1). Ten coastal villages from these areas were chosen as study sites. Aborlan, Puerto Princesa City and the rest of southern Palawan are vulnerable to sea level rise, whereas Taytay and the rest of northern Palawan are vulnerable to extreme heating events, unstable water supplies and sea level rise, according to the Department of Environment and Natural Resources (DENR) climate change exposure map (Fig. A1) [39]. Aborlan is also highly prone to landslides, while Puerto Princesa City has the highest population at risk from landslides and storm surges [40]. Both areas have mainstream economic activities, including airports, seaports, malls, schools and populated urban areas on the east coast, where storms make landfall first, making them more vulnerable to the effect of changing climate. Furthermore, the province of Palawan is the largest producer of seaweed in the country, and Taytay is one of the main producers in the province [41]. The recent onslaught of Typhoon Rai caused unprecedented losses to seaweed farmers, which environmentalists identified as an escalating issue fuelled by climate change [41]. Due to the vulnerability of the chosen study areas, they are ideally suited to explore how coastal communities perceive climate change and anthropogenic pressures that impact the coastal marine ecosystem.

The target populations were households within coastal marine areas in our three selected study areas, and the respondents were restricted to 18 years old and above. Literacy rates among the target populations were variable, which is why we decided to use a face-to-face survey, rather than self-completion. However, it was evident during the stakeholder workshops and discussions that they have good knowledge of the local environmental conditions and causes, so the topics of the survey were more familiar to them.

### Survey procedure

The survey was divided into four questions (see Appendix A). The first question aimed to understand if the participants believe that the climate in the locality was changing, using a semantic differential (bipolar) response rating scale with anchor points (1) ‘fully disagree’ to (7) ‘fully agree’. The second question sought to understand the participants’ observations and experiences of the various climate change impacts, using a semantic differential (bipolar) rating scale with anchor points (1) ‘very low’ to (7) ‘very high’. The third focused on perceived risks of climate change impacts on the coastal areas using a semantic differential (bipolar) rating scale with anchor points (1) ‘fully disagree’ to (7) ‘fully agree’, while the fourth question explored participants’ perceived risks of climate change impacts and anthropogenic pressures on coral reef and seagrass ecosystems, using a semantic differential (bipolar) rating scale with anchor points (1) ‘very low’ to (7) ‘very high’.

A two-stage pilot testing was conducted to ensure that participants would understand the questions. An in-home face-to-face survey was conducted using a Computer Assisted Personal Interviewing (CAPI) method, employing a tablet computer (Samsung Galaxy Tab A, Samsung Electronics Co., Ltd, Suwon-si, South Korea) with a pre-loaded questionnaire available in Filipino and English languages. The questionnaire was formatted using free data collection software (KoBo Toolbox v.2, Harvard Humanitarian Initiative, Cambridge, MA, USA).

The development of the survey was through a co-creation approach, with most of the content emerging from discussions and workshops with local stakeholders. The survey was drafted in line with the eDPSEEA model, which integrates human health and environmental impact on the ecosystem [36]. The finalised survey was very complex as it contained all aspects of the eDPSEEA model. In this study, the focus was only on climate change awareness and the perceived climate change risks in the coastal areas of Palawan.

### Data analysis

**Figure 2:**
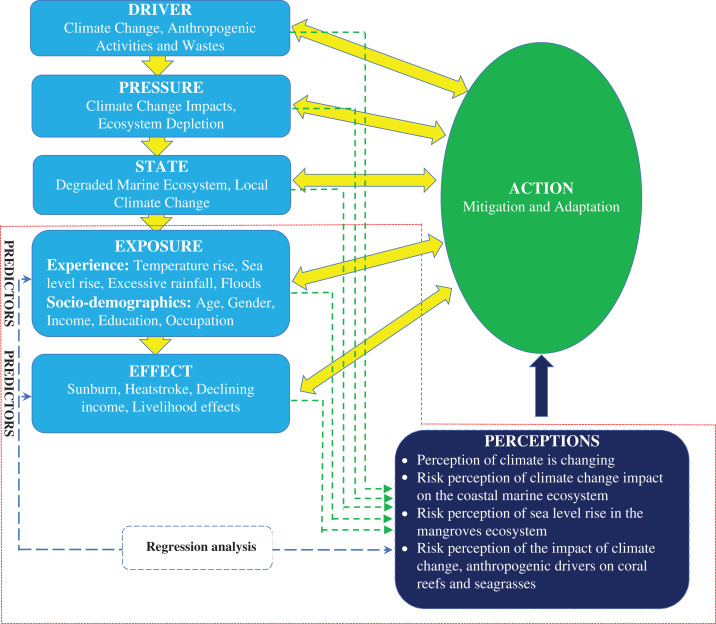
Conceptual framework used in data analysis of the relationship between predictors and risk perceptions of climate change impacts based on the eDPSEEA model. (Analysis is focused only on the highlighted, red-dotted line.) (Source: Authors, 2022.)

SPSS version 26.0 for Windows was used for all data analyses. The relationships analysed were the influence of the ‘Exposure’ and ‘Effect’ (as per the eDPSEEA model) on the perception of climate change impacts on the coastal communities (Fig. 2). Descriptive statistics (mean, standard deviation and standard error) were used to analyse and organise the characteristics of the data.

An exploratory factor analysis (EFA) using principal component analysis (PCA) was used to reduce data on risk perceptions of climate change impact in the coastal areas (six variables) and on the risk perceptions of factors affecting coral reefs and seagrass beds (17 variables), to a smaller set of summary variables (factors) and to explore the underlying theoretical structure relating to these perceptions (Tables A1 and A2) [42]. To confirm if PCA was suitable, the Kaiser–Meyer–Olkin (KMO) value was set at ≥ 0.70 to indicate good sampling adequacy, and Bartlett’s Test of Sphericity was set at *P* < 0.001 to confirm highly significant correlations among the variables [43,44]. The number of the retained factors was based on the criterion of the eigenvalue (>1.0) and examination of the scree plots. The retained factors underwent reliability analysis with Cronbach’s value set at α ≥ 0.70 to indicate good internal consistency [45]. Finally, we used linear regression to analyse the relationships between the risk perceptions of climate change impacts and the predictors [46]. The risk perceptions of climate change impacts based on PCA factoring will be the outcome variables, while the personal experiences of climate-related events and socio-demographic variables will be used as predictor variables (see Tables A1 and A2 for groupings).

On the risk perception of sea level rise impact on the mangrove areas, we used an additional test (paired samples t-test) to determine if the presence of mangroves compared to the absence of mangroves had a significant effect on risk perception of sea level rise impact. This was followed by calculating the effect size using Cohen’s d.

## Results

### Socio-demographics

A total of 291 respondents participated (Table 1) across ten barangays: two barangays in Aborlan, four in Taytay and four in Puerto Princesa City, with a higher number of females (59.1%) than males (39.5%). The higher percentage of female participants was in part due to the time of day the interviews were conducted (morning and afternoon), as many male household members would have left home for work at sea, as elaborated in another paper from the same survey [47].

**Table 1.** Socio-demographic characteristics of the respondents (n = 291)

Category	Aborlan (n = 61)		Puerto Princesa (n = 68)		Taytay (n = 162)		Total sample (n = 291)		
	n	%	n	%	n	%	n	%
**Gender**								
Female	33	54.1	44	64.7	95	58.6	172	59.1
Male	27	44.3	23	33.8	65	40.1	115	39.5
Missing data	1	1.5	1	1.5	2	1.2	4	1.4
**Income**								
Poor (< $196.70/month)	47	77.0	47	69.1	121	74.7	215	73.9
Not poor (≥ $196.70/month)	9	14.8	18	26.5	30	18.5	57	19.6
Missing data	5	8.2	3	4.4	11	6.8	19	6.5
**Age group**								
19–29	12	19.7	10	14.7	21	13.0	43	14.8
30–39	15	24.6	15	22.1	33	20.4	63	21.6
40–49	16	26.2	21	30.9	45	27.8	82	28.2
50–59	10	16.4	12	17.6	31	19.13	53	18.2
60–99	7	11.5	9	13.2	29	17.9	45	15.5
Missing data	1	1.6	1	1.5	3	1.9	5	1.7
**Education level**								
Elementary	32	54.2	32	47.8	55	35.3	119	42.2
High school	23	39.0	27	40.3	75	48.1	125	44.3
College	4	6.8	8	11.9	26	16.7	38	13.5
Missing data	2	3.3	1	1.5	6	3.7	9	3.1
**Occupation**								
Fisherfolk	53	86.9	57	83.8	142	87.7	252	86.6
Non-fisherfolk	5	8.2	10	14.7	15	9.3	30	10.3
Missing data	3	4.9	1	1.5	5	3.1	9	3.1

(Source: Authors, 2022.)

### Climate change awareness and personal experiences of impacts

**Figure 3:**
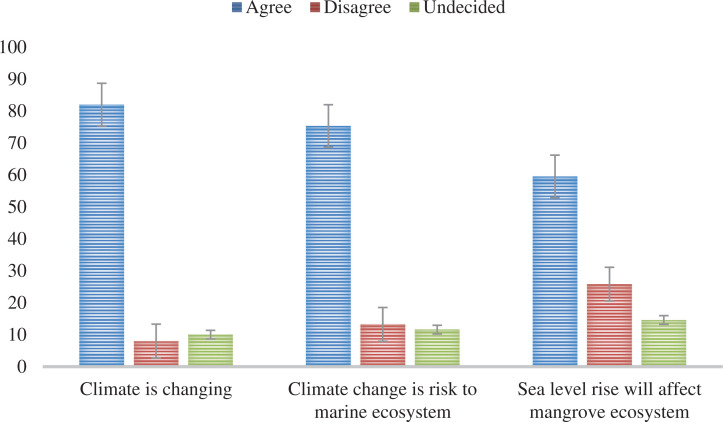
Proportion of participants who perceived that the climate in their locality is changing; climate change is a risk to the coastal marine ecosystem; and sea level rise will affect the mangrove ecosystem (n = 291). (Percentage was based on valid responses.) (Source: Authors, 2022.)

**Figure 4:**
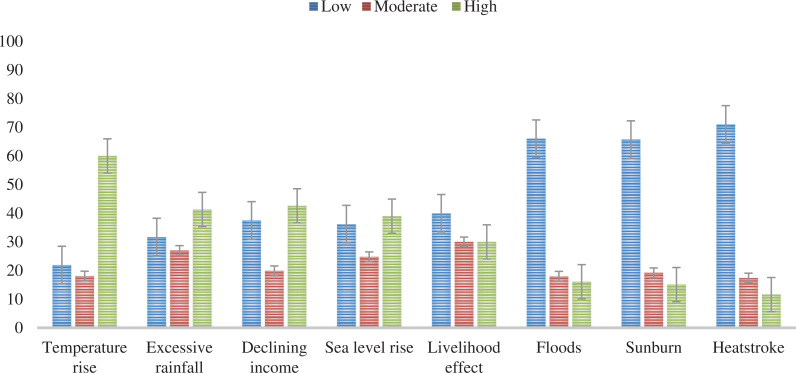
Proportion of participants who perceive low, moderate or high frequency of experience of various climate change impacts. The response options provided to the participants is a bipolar rating scale: 1 = very low to 7 = very high. Low category included scores 1–3, moderate category score 4, and high category scores 5–7 (n = 291). (Graph whiskers are standard error of the mean.) (Source: Authors, 2022.)

Most of the participants (82%) agreed that the climate in their locality is changing; 8% disagreed, while 10% were undecided (Fig. 3). The most common climate change impact experienced by a higher proportion of participants (60%) is local temperature rise (Fig. 4). Other climate change impacts that a higher proportion of the participants also experienced are excessive rainfall (41.2%), declining income (42.6%) and sea level rise (39%) (Fig. 4). In contrast, the climate change impacts that are less experienced by the higher proportion of the participants are flooding (66%), sunburn (65.7%), heatstroke (71%) and the effects on their livelihood (40%) (Fig. 4).

Their personal experiences with climate-related events and their awareness of climate change were also analysed. The results suggest that personal experiences with excessive rainfall (B = 0.17, *P* < 0.05) and local temperature rises (B = 0.17, *P* < 0.05) are significantly associated with a higher awareness of climate change (Table 2). Regarding socio-demographic factors, the 40–49 years old group (B = −0.69, *P* < 0.05) has significantly lower climate change awareness compared with the 19–29 years old group (Table 3).

**Table 2.** Results of linear regression model exploring the association between participants’ personal experience and climate change awareness; personal experience and risk perception of climate change impacts in the coastal marine ecosystem in Palawan, Philippines (standard errors in parenthesis)

Predictors (experiences)	Outcome variables (awareness and risk perceptions)		
	Climate change awareness	Risk perception of climate change impact on coastal marine ecosystem^1^	Risk perception of sea level rise impact on mangroves ecosystem^1^
Constant (B)	4.497 (0.40)***	4.733 (0.49)***	3.650 (0.43)***
**Local temperature rise**	0.17 (0.08)*	0.01 (0.09)	−0.03 (0.08)
**Sea level rise**	0.09 (0.07)	0.19 (0.09)*	0.36 (0.08)***
**Excessive rainfall**	0.17 (0.08)*	0.01 (0.10)	0.07 (0.09)
**Floods**	−0.06 (0.07)	−0.16 (0.08)	−0.03 (0.08)
**Heatstroke**	−0.01 (0.07)	−	−
**Sunburn**	0.06 (0.06)	−	−
**Declining income**	0.16 (0.09)	0.11 (0.10)	−0.14 (0.09)
**Livelihood effect**	−0.17 (0.09)	−0.13 (0.11)	0.03 (0.10)

***P < 0.001; *P < 0.05.

^1^Variable obtained from the data reduction method (PCA), see Table A1.

Note: Heatstroke and sunburn were used as predictors only in climate change awareness.

(Source: Authors, 2022.)

**Table 3.** Results of linear regression model exploring the association between participants’ socio-demographic characteristics and their awareness; socio-demographic characteristics and risk perceptions of climate change impacts in the coastal marine ecosystem in Palawan, Philippines (standard errors in parenthesis)

Predictors (experiences)	Outcome variables (awareness and risk perceptions)		
	Climate change awareness	Risk perception of climate change impact on coastal marine ecosystem^1^	Risk perception of sea level rise impact on mangroves ecosystem^1^
Constant (B)	6.429 (0.51)***	4.296 (0.57)***	4.467 (0.54)***
**Gender** (ref = male)	−	−	−
Female	−0.18 (0.22)	0.69 (0.25)**	0.25 (0.23)
**Education level** (ref = elementary)	−	−	−
High school	0.27 (0.24)	0.33 (0.26)	0.29 (0.25)
College	0.58 (0.33)	0.64 (0.37)	0.28 (0.34)
**Income** (ref = poor)	−	−	−
Not poor	0.28 (0.27)	0.59 (0.29)*	−0.24 (0.27)
**Occupation** (ref = non-fisherfolk)	−	−	−
Fisherfolk	−0.17 (0.36)	0.16 (0.41)	−0.42 (0.38)
**Age group** (ref = 19–29 years old)	−	−	−
30–39 years old	−0.36 (0.35)	−0.12 (0.39)	0.60 (0.36)
40–49 years old	−0.69 (0.33)*	−0.05 (0.37)	0.78 (0.34)*
50–59 years old	−0.16 (0.36)	0.57 (0.41)	1.40 (0.37)***
60–99 years old	−0.07 (0.38)	0.84 (0.43)	1.35 (0.41)***
**Study sites** (ref = Puerto Princesa)	−	−	−
Aborlan	−0.50 (0.32)	−0.15 (0.36)	−0.76 (0.33)*
Taytay	−0.27 (0.26)	0.01 (0.28)	−0.59 (0.27)*

***P < 0.001; *P < 0.05.

^1^Variable obtained from the data reduction method (PCA), see Table A1.

(Source: Authors, 2022.)

### Risk perception of climate change impact on the coastal marine ecosystem

The ‘climate change impact on the coastal marine ecosystem’ factor resulted from the PCA of two variables (Table A1). Most of the participants (75%) perceived that ‘climate change impact’ is a risk to the mangrove ecosystem as well as to the function and structure of the whole coastal marine ecosystem (Fig. 3). The personal experience of sea level rise (B = 0.19, *P* < 0.05) was the only significant risk perception predictor of the impact of climate change on the coastal marine ecosystem. Females were also found to have a higher risk perception (B = 0.69, *P* < 0.05) than males. Further, the not-poor group (B = 0.59, *P* < 0.05) had a significantly higher risk perception than the poor group. Other socio-demographic predictors did not show significant differences (Table 3).

### Risk perception of sea level rise impacts on the mangrove ecosystem

**Figure 5:**
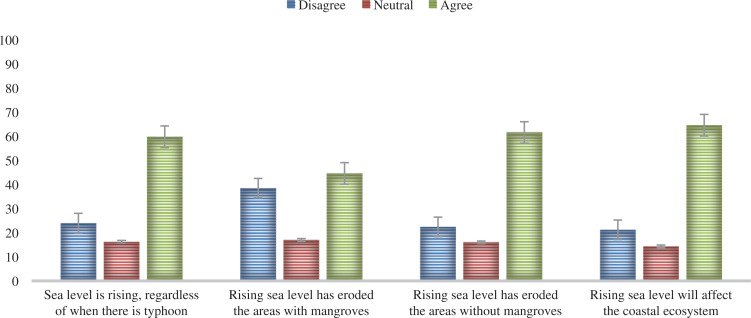
Proportion of participants’ risk perceptions on the individual variables regarding ‘sea level rise impacts’ (n = 291). (Source: Authors, 2022.)

The ‘sea level rise impact’ factor was a result of the PCA of four variables (Table A1). In general, the ‘sea level rise impact’ was perceived by many (59.5%) of the participants to cause coastal erosion to areas without mangroves and that it will affect the mangrove ecosystem, and the sea level is rising regardless of when there is a typhoon (Fig. 3). Analysis of the individual variables in the sea level rise impact showed that 60% agreed that the sea level was rising regardless of typhoon occurrence. Most participants also perceived that the sea level rise had eroded areas without mangroves (61.6%) and that it will affect the coastal ecosystem (64.6%). A considerable portion of the participants (44.6%) also perceived that sea level rise had eroded areas with mangroves (Fig. 5). The impact of sea level rise on coastal erosion based on the participants’ perception in areas with mangroves and without mangroves displayed a significant difference; t = −6.65, *P* < 0.001 (Table A3). Further, Cohen’s d value (d = 0.42) suggested a moderate effect size.

In our analysis, the personal experiences or observations of rising sea level was the strongest predictor of the risk perception of sea level rise impact (B = 0.36, *P* < 0.001). Furthermore, the 60–99 years old (B = 1.35, *P* < 0.05), the 50–59 years old (B = 1.41, *P* < 0.05) and the 40–49 years old groups (B = 0.78, *P* < 0.05) have a significantly higher risk perception than the 19–29 years old group (Table 3). The study site was also found to influence the risk perception of sea level rise impact as the Aborlan (B = −0.76, *P* < 0.05) and Taytay (B = −0.59, *P* < 0.05) participants have significantly lower risk perception as compared with Puerto Princesa City participants.

### Risk perceptions of the factors affecting the coral reefs and seagrass beds

**Figure 6:**
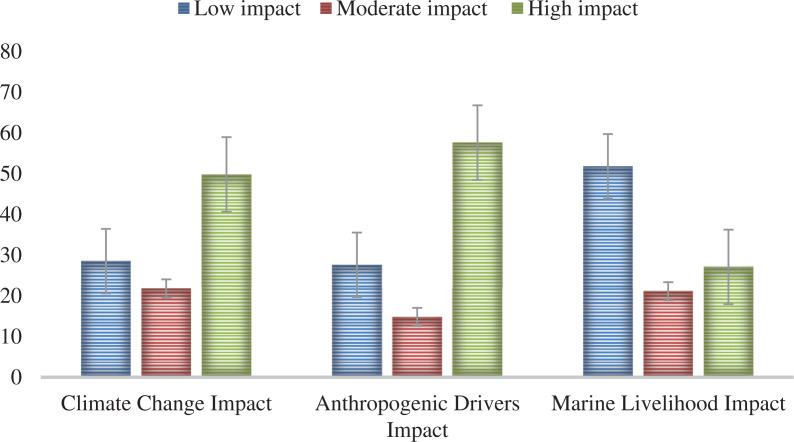
Proportion of participants who perceive low, moderate or high impacts to coral reefs and sea grass beds from different drivers. The response options provided to the participants is a bipolar rating scale: 1 = very low to 7 = very high. Low category included scores 1–3, moderate category score 4 and high category scores 5–7 (n = 291). (Source: Authors, 2022.)

Three factors affecting the coral reefs and seagrass beds were derived from PCA, namely: climate change impacts, anthropogenic pressures and marine livelihood (Table A2). Results showed that most of the participants perceived the anthropogenic pressures (57.6%) and climate change (50.3%) to have a high impact on the coral reefs and seagrass beds, while marine livelihood was perceived to have a low impact (51.8%) (Fig. 6).

The local temperature rise is a significant predictor of the perceived climate change impact (B = 0.16, *P* < 0.05), anthropogenic pressures (B = 0.25, *P* < 0.01) and marine livelihood impact (B = 0.21, *P* < 0.01) (Table 4). Additionally, excessive rainfall and declining income are perceived as significant risk predictors of climate change impact and anthropogenic pressures (Table 4).

**Table 4.** Results of linear regression predicting the participants’ risk perception of climate change impact, anthropogenic pressures and marine livelihood from their personal experiences of climate-related events in the coastal marine environment of Palawan, Philippines (standard error in parenthesis)

Predictor variables	Perceived impacts on corals reefs and seagrasses (outcome variables)		
	Climate change impact^1^	Anthropogenic drivers impact^1^	Marine livelihood impact^1^
Constant (B)	2.19 (0.36)***	2.27 (0.39)***	1.41 (0.46)**
**Local temperature rise**	0.16 (0.07)*	0.26 (0.08)***	0.21 (0.09)**
**Sea level rise**	0.08 (0.07)	−0.01 (0.07)	0.03 (0.09)
**Excessive rainfall**	0.19 (0.08)*	0.16 (0.08)*	0.09 (0.10)
**Floods**	−0.10 (0.06)	−0.08 (0.07)	−0.07 (0.08)
**Declining income**	0.30 (0.07)***	0.19 (0.08)*	0.09 (0.08)
**Livelihood effect**	−0.05 (0.08)	−0.01 (0.09)	0.08 (0.11)

*P < 0.05; **P < 0.01; ***P < 0.001.

^1^Variable obtained from the data reduction method (PCA), see Table A2.

(Source: Authors, 2022.)

On socio-demographic variables, the group categorised as ‘not poor’ have a significantly higher risk perception of climate change impact (B = 0.94, *P* < 0.001), anthropogenic pressures (B = 1.19, *P* < 0.001) and marine livelihood (B = 1.07, *P* < 0.001) compared to poor participants. The high school group (B = 0.51, *P* < 0.05) has shown a significantly higher risk perception of climate change impact compared with the elementary group. On the other hand, the 40–49 years old group (B = −0.73, *P* < 0.05) has also shown significantly lower risk perception compared with the 19–29 years old group.

## Discussion

The results from this study contribute to a greater understanding of the relationship between coastal community perceptions and climate change impacts, which in turn adds knowledge to the gaps about how to involve the public in building climate change resilience efforts.

### Role of personal experiences in shaping climate change awareness and risk perceptions

Climate change awareness and risk perceptions can be shaped by direct experiences of extreme weather events, local weather anomalies [48,49] and climate-related livelihood damage [50,51].

#### Experience with extreme weather events and anomalies

In our results, most study participants (82%) perceived that climate change was happening, and this is consistent with the results from a nationwide survey conducted in the Philippines in February 2021, which found that 83% of Filipinos believe the climate is changing [52]. Of the various extreme weather events and weather anomalies, the personal experience of temperature rise is the strongest predictor of climate change in Asian and African countries [27–29]. Our analysis showed that the personal experience of temperature rise was a significant predictor of climate change awareness. These findings are in line with more evidence suggesting that personal experiences of local weather anomalies (i.e., local temperature rises) and extreme weather events could influence perceptions and attitudes toward climate change [29,53–56]. This study also found that the person’s own experience with excessive rainfall is another significant predictor of climate change awareness. Excessive rainfall is an unusual occurrence in Palawan and may not significantly impact climate change perception because it cannot be easily recalled [48,57]. However, the excessive rainfall brought by typhoon Ketsana (2009) may have left a lasting impression. Previous study suggests that deviations from normal occurrences, such as excessive rainfall, may be perceived by locals as an indicator of climate change [58]. Other studies also show that experiences of climate-related events can generate climate change concern and awareness if they are: (1) unusual weather events compared to local historical events; and (2) they are associated with significant financial and/or personal damages [49–51]. This study was not able to capture the perceived impacts of the recent Typhoon Rai devastation in Palawan in the last quarter of 2021 which the researchers believe would be significant in changing the perception of those who are skeptical of climate change. Further research in relation to excessive rainfall, flooding and change in weather patterns is recommended in Palawan to promote climate change awareness and concern.

This study also analysed the relationship between personal experience of extreme weather events and anomalies with the perceived impacts of climate change, anthropogenic drivers and marine livelihood on the seagrasses and coral reefs (Table 4). The findings revealed that the perceived risks brought by anthropogenic drivers and climate change impacts were found to be significantly associated with personal experiences of local temperature rise and excessive rainfall. The findings also revealed that study participants perceived anthropogenic drivers to be the major factor damaging coral reefs and seagrasses. Climate change was also perceived to have a high impact on the coral reefs and seagrasses but to a lesser extent than anthropogenic drivers. The perception of the participants is in line with a previous study which revealed that anthropogenic drivers pose a far greater immediate threat to coral reefs than climate change [59]. It must be noted that anthropogenic drivers and climate change impacts are interconnected and that anthropogenic drivers are the reason why there are climate change impacts. Thus, the impact of both anthropogenic drivers and climate change must be viewed as per our conceptual framework (Fig. 2). Moreover, our findings that the experience of local temperature rise influences the perceived impacts of climate change and anthropogenic drivers is supported by the empirical evidence about the observed ocean temperature rise trend in different regions of the world [60]. The warmer temperatures can cause coral reefs to bleach and seagrasses to alter growth rates, resulting in reef fish deaths [61,62]. In addition, anthropogenic drivers result in the contamination of aquatic environments, which is one of the leading types of pollution that has significant negative impacts on coral reefs and seagrasses [63]. This study also found that personal experience of excessive rainfall is a significant predictor of the anthropogenic drivers and climate change impact on the seagrasses and coral reefs (Table 4). Excessive rainfall results in increased runoff of freshwater, sediment and land-based pollutants, which increase algal blooms and turbidity, thereby inhibiting light penetration that is necessary for the survival and growth of coral reef and seagrass ecosystems [64–66].

Personal experience of local temperature rise was also found to be significantly associated with the perceived marine livelihood impact on seagrasses and coral reefs. The warming of the oceans means fewer marketable fish species to catch, which in turn induces overfishing and illegal fishing activities [67,68]. These destructive fishing practices have been identified as the primary threat to coral reefs and the quality of the coastal marine environment [47,69]. On the other hand, long-term fish cage operations, if poorly located and managed, will result in the reduction of the abundance and diversity of benthic species and the degradation of the surrounding habitats [70,71].

Our findings suggest that the perceptions of the coastal residents are consistent with the established scientific information that anthropogenic drivers, climate change impacts and marine livelihoods significantly impact coral reefs and seagrasses. As these perceived impacts are significantly associated with local temperature rise and excessive rainfall, it is therefore suggested that when communicating climate change risks and mitigation measures to the coastal communities one should start with explaining the impacts of local temperature rise and excessive rainfall. In this way, the coastal people can easily relate to and understand the impacts of climate change. Giving coastal communities a high level of climate-relevant knowledge on the impact of climate change and anthropogenic drivers on corals and seagrasses is vital for preserving reef systems and accepting climate change policies [72]. Our results also open an exciting new avenue of study focused on what the coastal communities are doing to preserve reef ecosystems and how they are doing this. Specifically, on how they adapt and mitigate the impact of climate change and reduce anthropogenic drivers on the corals and seagrasses. Moreover, we suggest explanatory or applied scientific research to determine the impact of climatic and anthropogenic drivers on corals and seagrasses.

#### Experience with sea level rise

Climate change is a disaster risk driver and is perceived by the coastal residents (75%) in this study to impact the mangroves and the coastal marine ecosystem. This is higher public concern about the risk brought about by climate change compared to 67% in a nationwide survey in 2018 (Philippines) [73]. The higher climate change concern among the coastal community compared to the public can be attributed to the higher vulnerability of coastal areas to adverse impacts caused by climate stressors on their surroundings and livelihoods which shape people’s climate risk perception [74,75]. However, 25% of the participants are skeptical and do not consider climate change as a coastal risk driver. This could be attributed to the perception of some coastal communities that the land along the coastal margin will persist permanently, and that those living there will be safe from natural coastal hazards (apart from rare storm surge events) [76].

Personal experience of sea level rise was found to be significantly associated with climate change risk perception to the mangrove ecosystem and the marine coastal ecosystem, which is consistent with many studies that sea level rise is the main threat to the coastal ecosystem [7,15]. Our findings are also in line with earlier research that showed experience is one of the factors affecting how people perceive and respond to sea level rise impacts [48]. Notably, this study also found out that only 59.5% of the participants agree that sea level rise will cause coastal erosion and affect the coastal ecosystem (Fig. 3). The skepticism expressed by 40.5% of the participants that sea level rise will cause major damage to coastal areas could be attributed to the perception that mangroves can prevent coastal erosion (Fig. 5). Nationwide, the skeptical risk perceptions of sea level rise impact could be attributed to a lack of prominence given by the media outlets to the phenomenon [77]. Coastal residents in the Philippines tend to disregard the risk of sea level rise possibly because of their fisheries’ livelihood, causing them to generally prefer in situ adaptation strategies rather than relocation to the mainland [78]. This is in line with findings in a study conducted in the United States Gulf Region where public perceptions of sea level rise remain to be a temporally distant issue among coastal residents [79]. In contrast, research in New Zealand found that adults were overestimating the amount of sea level rise expected by 2100, which can result in feeling anxious rather than being motivated to mitigate and adapt [76]. Overestimation of sea level rise impact in New Zealand results from indiscriminate media reporting of the sea level rise warning that it could reach 5 m by 2100 [76].

The results revealed that 61.6% of the participants perceived that coastal areas without mangroves are eroded by sea level rise, compared to only 44.6% who perceived that areas with mangroves are also eroded, implying that most of them are aware of how important mangroves are to preventing coastal erosion (Fig. 5). This awareness can result in mangrove preservation for their protection. Our findings suggest the importance of training and communication tools to effectively relay information about coastal risks brought on by climate change and the impacts of sea level rise to help motivate coastal residents to act. By educating coastal communities about the importance of mangrove preservation and building their capacity to manage mangrove forests sustainably, climate-friendly policies were more likely to be supported [9,59].

#### Experience with climate-related livelihood damages

Coastal and low-income communities are most vulnerable to climate change impacts [61,80]. Our results showed that participants perceive that declining income is the strongest predictor of climate change and anthropogenic drivers’ impact on seagrasses and coral reefs (Table 4). The impact is already felt by fishers by them getting lower revenue, which creates a domino effect of several other socio-economic consequences including low economic standing, non-existent social welfare or pension systems for fishers, and poor health and living standards for their families [61,81]. Fisherfolk perceived that loss of income was a result of climate change impacts such as rising sea levels, excessive rainfall, temperature rise, the decline in fish catch and loss of coral reefs and seagrass cover [61]. Additionally, anthropogenic drivers also result in damaging the coral reefs and seagrass meadows, thereby reducing seaweed farmers’ and fisherfolk’s incomes [63,69]. It is therefore necessary for these vulnerable fishers in the coastal areas to acquire different adaptation and coping strategies to mitigate these impacts [82,83]. To enhance their resilience to the impacts, fishers need development assistance that protects their well-being, prioritises alternative livelihoods and provides technical skills training [61,84,85]. Additionally, the coastal community must support the preservation of mangroves, seagrass and coral reefs, which provide a habitat for important commercial and recreational species and stabilise the seafloor [61,86,87].

### Role of socio-demographic factors in shaping climate change awareness and risk perceptions

Understanding population demographics and heterogeneity is essential for improving our understanding of climate change and risk perceptions of the impacts. Our results showed that age, educational attainment, household income and study sites influence the climate change awareness and risk perceptions of the participants.

#### Gender

The results showed that women have a higher risk perception of climate change’s impact on the coastal marine ecosystem than men. This is consistent with findings that women consistently have a higher risk perception and express slightly greater concern about climate change compared to men [88,89]. Women tend to have a higher sensitivity to environmental concerns compared to men due to their higher levels of socialisation, rich local social networks and being more socially responsible [90,91]. The gender gap in perceiving climate change has not changed much since 2010, even though men’s understanding of the scientific consensus has improved over time [92]. The United Nations has recognised that the climate change crisis is not ‘gender neutral’ because women are more vulnerable to its effects than men, primarily because they make up the majority of the world’s poor and are more dependent on natural resources for their survival, which is under threat from climate change [93,94]. Therefore, it is imperative that policymakers should aim to advance the implementation of gender-responsive climate policies and mandates across all areas of discussion when taking actions to mitigate the impact of climate change [95].

#### Educational attainment

**Figure A2:**
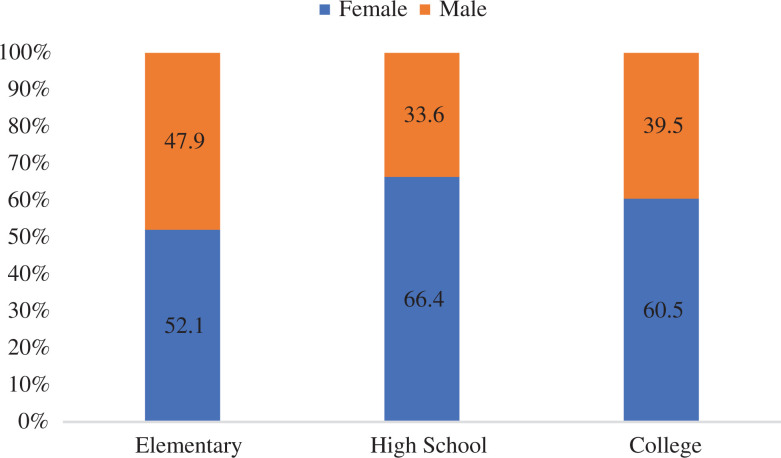
Cross-tabulation count of participants’ gender and educational attainment who perceived the climate change impact on coral reefs and seagrasses (n = 291). (The count was based on listwise deletion.) (Source: Authors, 2022.)

Climate change’s impact on coral reefs and seagrasses is perceived differently depending on educational attainment, in line with previous studies which showed that those with higher education tend to have more concern for the environment [96,97]. Surprisingly the high school category has a slightly higher risk perception than the college category, although they are not significantly different at *P* < 0.05. The slight difference could be attributed to the fact that there is a higher ratio of women to men among the college group (65%) compared to the high school group (51%) (see Fig. A2). A previous study showed that women’s self-perceived knowledge is higher than men’s among people with low levels of education but higher for men among people with high levels of education [98]. It should be noted, however, that our study had some gender imbalances, so we should be cautious when interpreting the interaction between gender and education results.

#### Income

Poor households have a significantly lower risk perception of climate change’s impact on the coastal marine ecosystem (Table 3), which is in line with another study conducted in Singapore which found that low-income households reported a lower level of knowledge compared with higher-income households [99]. Poor households also have significantly lower risk perceptions of the impact of climate change, anthropogenic pressures and marine livelihood on sea grasses and coral reefs compared with not-poor households (Table 5). Lower climate change risk perception for poor households compared to not-poor households could be explained by the fact that low-income households and communities develop academic skills at a slower rate than those from higher-income groups [99]. Poverty levels are strongly linked to educational attainment. In the Philippines, the heads of two of three poor households have only reached elementary education and below [100]. Further, the lack of economic resources was a major barrier to paying attention to climate change, as they had more pressing priorities, such as the financial pressure of daily living [101]. For poor households who face more financial pressure than high-income households, climate change is less likely to be a concern.

**Table 5.** Results of linear regression analysis predicting the participants’ risk perceptions of climate change impact, anthropogenic pressures and marine livelihood from key socio-demographic characteristics in the coastal marine environment of Palawan, Philippines (standard error in parenthesis)

Predictor variables	Perceived impacts on corals reefs and seagrasses (outcome variables)		
	Climate change impact^1^	Anthropogenic drivers impact^1^	Marine livelihood impact^1^
Constant (B)	3.658 (0.48)***	4.162 (0.52)***	3.862 (0.56)***
**Gender** (ref = male)	−	−	−
Female	0.27 (0.21)	0.22 (0.22)	0.10 (0.25)
**Education level** (ref = elementary)	−	−	−
High school	0.51 (0.22)*	0.14 (0.24)	−0.14 (0.27)
College	0.30 (0.33)	0.23 (0.35)	0.18 (0.39)
**Income** (ref = poor)	−	−	−
Not poor	0.94 (0.24)***	1.16 (0.26)***	1.073 (0.30)***
**Occupation** (ref = non-fisherfolk)	−	−	−
Fisherfolk	0.20 (0.35)	0.08 (0.37)	−0.28 (0.42)
**Age group** (ref = 19–29 years old)	−	−	−
30–39 years old	−0.40 (0.33)	−0.43 (0.35)	−0.62 (0.39)
40–49 years old	−0.40 (0.31)	−0.52 (0.33)	−0.73 (0.36)*
50–59 years old	−0.41 (0.34)	−0.52 (0.37)	−0.38 (0.41)
60–99 years old	−0.19 (0.37)	−0.42 (0.40)	0.04 (0.44)
**Study sites** (ref = Puerto Princesa)	−	−	−
Aborlan	0.14 (0.30)	0.53 (0.33)	0.55 (0.37)
Taytay	0.29 (0.24)	0.46 (0.26)	0.52 (0.29)

*P < 0.05; **P < 0.01; ***P< 0.001.

^1^Variable obtained from the data reduction method (PCA), see Table A2.

(Source: Authors, 2022.)

#### Age

The 19–29 years old group has higher climate change awareness and risk perception of marine livelihood impact on coral reefs and seagrasses compared with other age groups (see Tables 3 and 5), in line with other studies that report the younger generation in the USA worries more about the effects of global warming than the older generation [32]. In contrast, for the risk perception of sea level rise impact on the mangrove ecosystem, the older generations group (40–49 years old and older) was found to have the higher risk perception compared with the 19–29 years old group (Table 3). Scientific knowledge about the causes, impacts and solutions to climate change generally increases with age, as would be expected with increased scientific education and exposure to information [102]. Having lived many years and experienced the various changes that have taken place in coastal areas, the older generation may have acquired enough wisdom or experienced enough changes in their youth to know about the threat that climate change poses [102]. This could be because younger generations have less experience and exposure to the impact of rising sea levels and as older generations have more experience, they perceive greater damage caused by sea level rise compared with younger generations.

#### Location

Puerto Princesa City participants have higher climate change awareness compared with Aborlan and Taytay participants (Table 3). These results suggest that climate change awareness might be influenced by geographical context [33,103]. The differences in climate change awareness could be attributed to the more publicised people’s participation in the reforestation of mangroves in Puerto Princesa which has been going on for more than two decades and resulted in the planting of millions of mangrove trees, thereby increasing beach coverage [103–105]. In Aborlan and Taytay, which are more rural than Puerto Princesa, there are fewer environmental conservation activities publicised and participants have limited media coverage of those activities, which may explain the lower climate change awareness [105,106]. Similarly, Puerto Princesa participants have significantly lower risk perception (*P* < 0.05) of the impact of marine livelihood on the sea grasses and coral reefs as compared with Aborlan participants (Table 5). Further studies are necessary to conclude a causal association between the differences in perceptions.

### Limitations

The findings of this study must be seen considering some limitations. The first is that we did not include in this study questions about how they perceived the impact of climate change on their livelihood and food security. These additional factors could be significant in determining how people perceive the overall impact of climate change, which will help communities and policymakers to develop more environmentally sustainable and socially adaptable programmes. However, we intend to address these limitations in future studies.

The second limitation concerns the state of ecosystems impacted by climate change in the coastal areas. Directly cross-verifying the state of ecosystems impacted by climate change and the historical data of climate-related events in the coastal areas compared to their perceptions would give a good measurement of their current level of climate-relevant knowledge. Nevertheless, their perceptions are useful in understanding their mental model. Furthermore, this limitation is another avenue for potential future research.

## Conclusions

As the impacts of climate change are likely to worsen the problems in vulnerable coastal areas, it is important to understand how experiences of climate-related events and various socio-demographic characteristics of the coastal community shape their awareness and risk perceptions. This study suggests that while coastal communities in our study sites have a high awareness (82%) of climate change, the remaining 18% are still notably unaware that climate change is happening. The most common climate change impacts observed or experienced by the participants are temperature rise and excessive rainfall. In descending order, other impacts of climate change experienced or observed by the participants in low frequency include declining income, sea level rise, floods, sunburn and heatstroke. Among these climate change experiences, temperature rise and excessive rainfall are significant predictors of climate change awareness.

Experience or observation of sea level rise is a significant predictor of risk perception of climate change impacts on the mangroves and coastal marine ecosystem. This study also established that ‘women’ and ‘not poor’ participants perceived the risk of climate change to the coastal marine ecosystem as higher compared to the reference groups. Furthermore, the 19–29 years old group has higher climate change awareness and more concern about marine livelihood impact on coral reefs and seagrasses compared with other age groups. In contrast, the 19–29 years old group has lower risk perception compared with older age groups in the risk perception of sea level rise impact on the mangrove ecosystem. Moreover, the risk perception of sea level rise impact is influenced by geographical context.

Most participants perceived that anthropogenic drivers and climate change have a high impact on the coral reefs and seagrasses, while marine livelihood is perceived as having a low impact. Local temperature rise, excessive rainfall and declining income are significant predictors of these risk perceptions. Education has a significant influence on the risk perception of the impact of climate change on coral reefs and seagrasses. While the ‘not poor’ participants have significantly higher risk perception compared to the ‘poor’ group in perceiving the impact of the various factors affecting coral reefs and seagrasses.

Future research on climate change mitigation should focus on how to improve the coastal community’s awareness and increase their willingness to support climate-friendly policies. There is a need for a bespoke climate change ‘knowledge management system’ and risk communication tools for different demographics to further increase awareness and concern for a healthy and sustainable coastal community. By addressing these issues from an interdisciplinary perspective, we can build adaptive capacity and reduce the vulnerability of coastal communities.

## Data Availability

The datasets generated during and/or analysed during the current study are available from the corresponding author on reasonable request.

## Appendix

### Appendix A

#### Questionnaire

**Table A1.** Perceptions of climate change impact in the coastal areas. The response options provided to the respondents was a bipolar rating scale: 1 = fully disagree to 7 = fully agree. n = 291

Perceptions	Responses (%)							Missing (%)	Mean	SD	Loadings
	1	2	3	4	5	6	7				
**Climate change impact on coastal marine ecosystem** ^1^	9.5	0.8	2.9	11.6	14.9	21.1	39.3	16.8	**5.41** ^a^	1.87	
Climate change is a threat to the mangroves	12.8	1.7	2.1	16.7	15.8	16.2	34.6	19.6	5.08	2.00	0.94
Climate change is a threat to the coastal ecosystem	10.5	1.3	0.4	12.2	11.8	18.1	45.8	18.2	5.51	1.92	0.94
**Sea level rise impact on mangroves ecosystem** ^1^	11.2	5.2	9.4	14.6	14.2	22.8	22.5	8.2	**4.74** ^a^	1.97	
Sea level is rising, regardless of when there is typhoon	18.0	1.5	4.5	16.2	9.8	12.0	38.0	8.6	4.86	2.24	0.79
Rising sea level has eroded the areas with mangroves	30.4	1.6	6.5	17.0	9.7	13.0	21.9	15.1	4.00	2.34	0.72
Rising sea level has eroded the areas without mangroves	15.6	2.0	4.8	16.0	11.2	13.2	37.2	14.1	4.94	2.16	0.86
Rising sea level will affect the coastal ecosystem	15.1	1.6	4.5	14.3	13.9	17.6	33.1	15.8	4.95	2.10	0.79

^a^Factor means.

^1^Factor obtained from data reduction through PCAs of perceived climate change impact in the coastal areas.

(Source: Authors, 2022.)

**Table A2.** Perceptions of factors affecting the coral reefs and seagrass beds. The response options provided to the respondents was a bipolar rating scale: 1 = fully disagree to 7 = fully agree. n = 291

Factors	Responses (%)							Missing (%)	Mean	SD	Loadings
	1	2	3	4	5	6	7				
**Climate change impacts** ^1^	9.2	5.9	13.4	21.8	24.7	14.2	10.9	17.9	**4.28** ^a^	1.53	
Temperature rise	13.0	3.5	11.3	18.6	26.8	12.1	14.7	20.6	4.38	1.84	0.73
Excessive rainfall	16.6	4.7	9.4	26.0	21.3	12.8	9.4	19.2	4.06	1.83	0.76
El Niño (drought)	13.4	3.9	7.8	22.1	22.1	15.6	15.2	20.6	4.43	1.87	0.79
Frequent typhoons	11.6	4.7	12.5	19.4	21.1	17.7	12.9	20.3	4.38	1.82	0.72
Runoffs	12.9	4.4	12.4	22.7	21.8	12.4	13.3	22.7	4.27	1.82	0.63
Natural calamities	15.3	4.8	10.5	16.7	18.7	21.1	12.9	28.2	4.33	1.94	0.42
**Anthropogenic drivers** ^1^	9.5	7.1	11.0	14.8	18.6	23.3	15.7	27.8	**4.52** ^a^	1.60	
Sewerage	12.3	4.8	8.3	14.0	23.7	18.0	18.9	21.6	4.61	1.92	0.72
Pollution	9.9	4.5	4.9	13.9	22.0	20.6	24.2	23.4	4.92	1.87	0.79
Domestic wastes	8.3	3.0	7.8	13.5	25.7	21.3	20.4	21.0	4.91	1.76	0.67
Land use change	25.9	6.5	6.5	20.4	17.9	10.0	12.9	30.9	3.80	2.09	0.64
Urbanisation	20.7	6.1	8.9	15.0	23.0	14.1	12.2	26.8	4.05	2.02	0.66
Illegal fisheries	8.6	3.6	5.0	5.4	15.3	25.7	36.5	23.7	5.38	1.88	0.71
**Marine livelihood** ^1^	31.2	11.9	8.7	21.1	13.8	9.6	3.7	25.1	**3.22** ^a^	1.74	
Pearl farms	41.9	5.8	6.8	22.0	6.3	6.8	10.5	34.4	3.07	2.12	0.82
Fish cages	31.5	8.0	9.0	31.0	9.0	5.5	6.0	31.3	3.19	1.86	0.83
Shellfish farms	44.8	4.4	4.4	26.5	4.4	6.1	9.4	37.8	2.97	2.09	0.90
Tourism related development	28.2	4.8	8.6	28.7	12.9	10.0	6.7	28.2	3.50	1.93	0.47

^a^Factor means.

^1^Factor obtained from data reduction through PCA of perceived impact of factors affecting the coral reefs and seagrass beds.

(Source: Authors, 2022.)

**Table A3.** Paired samples t-test comparing sea level rise impact on coastal erosion when there are mangroves compared to when there are no mangroves in the coastal areas of Palawan, Philippines

Pair variable	Mean	SD	SE	95% CI of the difference		t	DF	p
				Lower	Upper			
Rising sea level has eroded the areas with mangroves	3.99	2.34	0.15					
Rising sea level has eroded the areas without mangroves	4.94	2.16	0.14					
Pair: w/mangroves – w/o mangroves	−0.95	2.24	0.14	−1.23	−0.67	**−6.65*****	244	0.000

Note: Cohen’s d = 0.42; *** *P* < 0.001.

(Source: Authors, 2022.)
